# Anti-biofilm Activity of Povidone-Iodine and Polyhexamethylene Biguanide: Evidence from In Vitro Tests

**DOI:** 10.1007/s00284-023-03257-5

**Published:** 2023-04-01

**Authors:** Luc Gryson, Sylvie Meaume, Ina Feldkaemper, Filippo Favalli

**Affiliations:** 1 Belgian Defence, Medical Component, Brussels, Belgium; 2 ZoWe Nursing School, Brugge, Belgium; 3 Department of Geriatrics and Wound Care Unit, Hospital Rothschild, Assistance Publique Hôpitaux de Paris, Sorbonne Université, Paris, France; 4 Meda Pharma GmbH & Co. KG, a Viatris Company, Bad Homburg, Germany; 5 Meda Pharma S.p.A., a Viatris Company, Monza, Italy

## Abstract

Biofilm in chronic wounds is associated with delayed healing and ineffective local treatment. The purpose of this study was to investigate the in vitro anti-biofilm activity of two commonly used antimicrobials, povidone-iodine (PVP-I) and polyhexamethylene biguanide (PHMB). The rate of anti-biofilm activity of PVP-I, PHMB, and phosphate-buffered saline (negative control) was assessed on monomicrobial biofilms of varying maturity and composition. Antimicrobial efficacy was determined by counting colony-forming units (CFU). Live/dead cell staining and time-lapse confocal microscopy were also performed. Both PVP-I and PHMB demonstrated robust in vitro anti-biofilm activity against all tested biofilms; however, PVP-I had a more rapid action versus PHMB against methicillin-resistant *Staphylococcus aureus* (MRSA) biofilms, as determined by both CFU counts and microscopy. PVP-I completely eradicated *Pseudomonas aeruginosa* biofilms of 3- and 5-day maturity (in ≤0.5 h) and 7-day maturity (in ≤3 h), while PHMB only partially depleted cell density, with no complete biofilm eradication even after 24 h. In conclusion, PVP-I had a similar in vitro anti-biofilm activity to PHMB against biofilms of varying microbial compositions and maturity, and in some cases demonstrated more potent and rapid activity versus PHMB. PVP-I may be particularly effective in treating MRSA biofilms. However, further high-quality clinical research on the efficacy of antimicrobials is required.

## Introduction

Wound healing is a complicated, highly regulated process, crucial for restoring normal skin barrier function and preventing further damage [1, 2]. Many factors can lead to inadequate wound healing [2]; for example, the presence of a pathogenic biofilm [3–6]. A biofilm is a functional structure comprised of microbial cells attached to a surface and embedded in self-produced extracellular polymeric substances (EPS) [4]. Almost 80% of chronic wounds contain biofilms [7], and their presence is associated with prevention of wound healing and inefficient local treatment of the infection [3–6].

Biofilms exhibit high tolerance to antibiotics and antimicrobials and an ability to evade host defenses [3, 8]. Consequently, chronic wounds with biofilms (or ‘critically colonized’ wounds) are slow to heal and pose a significant burden on patient quality of life and cost of medical care [5, 8]. Early recognition of the biofilm stage of bacterial infection and rapid intervention with appropriate treatment is essential to enhance clinical outcomes [9].

A range of antimicrobials are used in the field of wound care, including povidone-iodine (PVP-I), polyhexamethylene biguanide (PHMB), octenidine, and sodium hypochlorite [8, 10, 11]. PHMB is widely recognized as an appropriate antimicrobial for use in critically colonized wounds and chronic wounds due to its broad spectrum of antimicrobial activity, tissue compatibility, capability of binding to an organic matrix, and wound-healing properties [10, 12]. Iodine-based antimicrobials, such as PVP-I, are also recommended for treatment of wounds with biofilm [8, 11, 13] and likewise possess favorable characteristics: a broader spectrum of antimicrobial activity compared with several antiseptics, including PHMB [14]; no reported bacterial resistance or cross-resistance to antibiotics or other antimicrobials; and an ability to promote wound healing [8].

Several studies have demonstrated the anti-biofilm activity of PVP-I in vitro. When the activity of several antimicrobials was tested against biofilm communities, the largest reduction in bacterial count was seen with PVP-I (57%), followed by PHMB (44%), and silver acetate (27%) [15]. PVP-I also demonstrated greater efficacy in reducing mixed *Pseudomonas* and *Staphylococcus* biofilms compared with antibiotics and silver-containing dressings [16], and significantly inhibited biofilm formation by *Staphylococcus epidermidis* and *Staphylococcus aureus*, even at sub-inhibitory concentrations [17]. PVP-I demonstrated rapid and long-lasting activity against microbial biofilms, mediating complete eradication of both *S. aureus* and *Pseudomonas aeruginosa* biofilms after 15 min exposure [18].

Based on this evidence, this study investigates the in vitro activity of PVP-I and PHMB against biofilms of clinically relevant microbes at varying levels of maturation.

## Materials and Methods

Four separate tests (Tests A–D) were performed to assess the in vitro activity of PVP-I and PHMB. In each test, the rate of anti-biofilm activity of 10% PVP-I, 0.1% PHMB (comparator), and phosphate-buffered saline (PBS, negative control) was assessed on biofilms of varying compositions and maturity.

### Microbial Isolates

All microbial isolates were obtained commercially. Manufacturer-stated isolation sources were as follows: ‘water bottle in animal room’ (*P. aeruginosa* ATCC 15442); ‘lesion’ (*S. aureus* ATCC 6538); ‘blood from a patient’ (*Enterococcus faecalis* ATCC 700802 [vancomycin resistant]); ‘hospital’ (*S. aureus* ATCC BAA-43 [MRSA]); and ‘man with bronchomycosis’ (*Candida albicans* ATCC 10231).

### Tests and Objectives

#### Test A: Microbial Screening

The objective of Test A was to determine and compare the rate of anti-biofilm activity of each of the three test solutions (10% PVP-I, 0.1% PHMB, and PBS) on biofilms of 2-day maturity. Monomicrobial biofilms of five different microbes were selected for screening based on their clinical relevance: *P. aeruginosa* ATCC 15442, *S. aureus* ATCC 6538, *Enterococcus faecalis* ATCC 700802 (vancomycin resistant), *S. aureus* ATCC BAA-43 (MRSA), and *Candida albicans* ATCC 10231. The anti-biofilm activity of the test solutions was assessed using the Minimum Biofilm Eradication Concentration (MBEC) Assay^®^ for all biofilms except the *C. albicans* biofilm. The MBEC Assay is a standardized model for studying biofilms [19], suitable for most microbes; however, it has a limited nutritional supply, constraining the growth of the biofilm. Robust *C. albicans* biofilm growth cannot be obtained with the MBEC Assay; therefore, this microbe was tested using the Centers for Disease Control (CDC) Biofilm Reactor^®^ model.

#### Test B: Mature *P. aeruginosa* Challenge

The objective of Test B was to determine and compare the rate of anti-biofilm activity of the test solutions on *P. aeruginosa* ATCC 15442 biofilms of 3-day, 5-day, and 7-day maturity. Since more mature biofilms require longer duration of growth and therefore greater nutrition, the CDC Biofilm Reactor model was used.

#### Test C: Mature *S. aureus *ATCC 6538 and *S. aureus* ATCC BAA-43 (MRSA) Challenge

The objective of Test C was to determine and compare the rate of anti-biofilm activity of the test solutions on *S. aureus* ATCC 6538 and *S. aureus* ATCC BAA-43 (MRSA) biofilms of greater maturity than Test A (3-day maturity) using the CDC Biofilm Reactor model.

#### Test D: Visualization

The objective of Test D was to visualize the anti-biofilm activity of the three test solutions on *S. aureus* ATCC BAA-43 (MRSA) biofilms of 2-day maturity using live/dead cell staining and time-lapse confocal microscopy.

### MBEC Assay

Overnight cultures of *P. aeruginosa* ATCC 15442, *S. aureus* ATCC 6538, *S. aureus* ATCC BAA-43 (MRSA), and *E. faecalis* ATCC 700802 (all cultured for Test A), adjusted to 1 × 10^5^ colony-forming units (CFU)/mL, were used to inoculate wells of a 96-well plate; one plate per microbial biofilm was cultured, and each test well was reproduced in triplicate. Peg lids were placed onto the 96-well plates, which were then incubated in tryptic soy broth (TSB) at 37 °C and 125 rpm for 48 h, with the TSB being replaced after 24 h. Following 48 h incubation, a biofilm growth check was performed to confirm growth of the biofilm on the pegs.

The challenge plate was set up with 200 μL/well of either 10% PVP-I, 0.1% PHMB, or PBS. The peg lid containing the biofilm was transferred to a rinse plate containing 200 μL/well PBS for 10 s before transferring it to the challenge plate. The challenge plate was incubated at room temperature for either 0.5, 3, 6, or 24 h, depending on the contact time specified. Following treatment, each peg lid was transferred to a recovery plate containing broad-spectrum neutralizer 1 (BSN 1; 200 μL/well).

### CDC Biofilm Reactor Model

Overnight cultures of *C. albicans* ATCC 10231 (cultured for Test A), *P. aeruginosa* ATCC 15442 (cultured for Test B), and *S. aureus* BAA-43 (MRSA) and *S. aureus* ATCC 6538 (both cultured for Test C) were used to inoculate the CDC Biofilm Reactor. The CDC Biofilm Reactor was incubated in batch phase on a magnetic stir plate for 24 h and then switched over to continuous phase. Nutrient flow tubing was attached to a carboy containing the relevant culture medium (TSB, or sabouraud dextrose broth for *C. albicans*), passed through a peristaltic pump and attached to the nutrient port on the top of the CDC Biofilm Reactor. The CDC Biofilm Reactor was then incubated in continuous phase for the remaining time required (24 h for 2-day biofilms, 48 h for 3-day biofilms, 96 h for 5-day biofilms, and 144 h for 7-day biofilms).

After the required time in continuous phase, the coupons were placed into individual 12-well plates and incubated with 4 mL/well of either 10% PVP-I, 0.1% PHMB, or PBS for either 0.5, 3, 6, or 24 h at room temperature, depending on the specified contact time. Each test was performed in triplicate. The following day, coupons were removed from the wells and added to 10 mL BSN 1.

### Colony-Forming Unit Counts

Following neutralization with BSN 1, all samples were sonicated at full power for 30 min. Each sample was serially diluted 1:10 in PBS, and dilutions were plated onto trypticase soy agar. Plates were incubated at 37 °C overnight and, the following day, CFUs were counted as a measure of cell density.

### Statistical Methodology

Raw data were entered into Microsoft Excel, and average CFU/mL was calculated. The Wilcoxon–Mann–Whitney test was used to make pairwise comparisons between the three treatments at each respective timepoint, to determine: (i) if treatment with the two antimicrobial agents, PVP-I and PHMB, was statistically superior to treatment with the negative control (PBS), and (ii) if treatment with PVP-I was statistically superior to treatment with the comparator (PHMB). JMP^®^ v15.0.0 (SAS Institute Inc.) software was used for all statistical analyses. Statistical superiority was defined as *P* < 0.05.

### Cell Staining and Confocal Microscopy

A chamber slide was inoculated with an overnight culture of *S. aureus* BAA-43 (MRSA) and then incubated at 37 °C and 125 rpm for 48 h. After 48-h incubation, cells were stained using the LIVE/DEAD™ *Bac*Light™ bacterial fluorescent staining kit. The LIVE/DEAD *Bac*Light kit contains two fluorescent stains, SYTO 9^®^ green-fluorescent nucleic acid stain, and propidium iodide red-fluorescent nucleic acid stain. When used together, bacterial cells with intact membranes stain green and bacterial cells with damaged membranes stain red.

A series of confocal fluorescence images were acquired with a LSM 780 Zeiss confocal microscope with a 40× (0.9 numerical aperture) air objective; visualization was carried out at 37 °C using an incubation chamber enclosing the microscope stage and body. Imaging was carried out in real time over 6 h.

Image processing was carried out using Fiji—ImageJ software, and the images were presented as a composite of the SYTO 9 green-fluorescent and propidium iodide red-fluorescent images.

## Results

### Test A: Anti-biofilm Activity of 10% PVP-I and 0.1% PHMB Against 2-Day Biofilms of *P. aeruginosa* ATCC 15442, *S. aureus* ATCC 6538, *E. faecalis* ATCC 700802 (Vancomycin Resistant), *S. aureus* ATCC BAA-43 (MRSA), and *C. albicans* ATCC 10231

Both PVP-I and PHMB significantly reduced bacterial cell density compared with the negative control for all microbes at most timepoints.

**Fig. 1:**
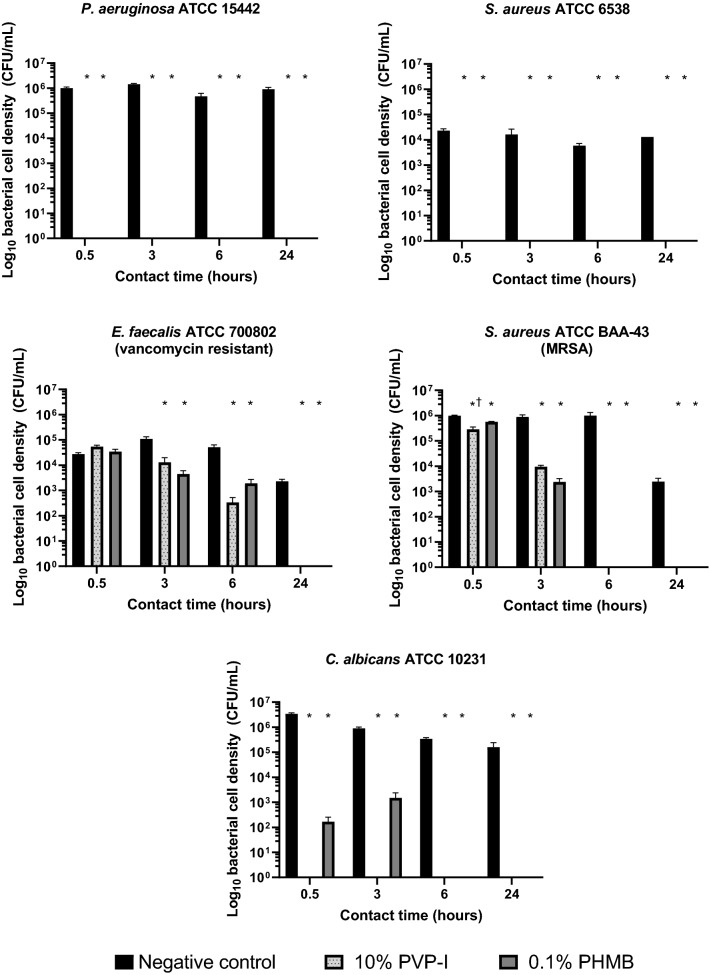
Bacterial cell density following treatment with PVP-I and PHMB in 2-day biofilms of *P. aeruginosa* ATCC 15442, *S. aureus* ATCC 6538, *E. faecalis* ATCC 700802 (vancomycin resistant), *S. aureus* ATCC BAA-43 (MRSA), and *C. albicans* ATCC 10231. Log_10_ bacterial cell density (CFU/mL) of 2-day biofilms following 0.5, 3, 6, and 24-h treatment with 10% PVP-I and 0.1% PHMB (*n* = 3). Error bars represent standard error of the mean. *Significant reduction (*P* < 0.05) versus negative control. ^†^Significant reduction (*P* = 0.0383) versus 0.1% PHMB. *CFU* colony-forming unit; *MRSA* methicillin-resistant *S. aureus*; *PHMB* polyhexamethylene biguanide; *PVP-I* povidone-iodine

PVP-I anti-biofilm activity was similar to PHMB activity across microbes. Both PVP-I and PHMB demonstrated fast (≤0.5 h), potent anti-biofilm activity against *P. aeruginosa* ATCC 15442 and *S. aureus* ATCC 6538 2-day biofilms, with complete eradication of the biofilm (CFU count of 0) after 0.5 h and sustained up to 24 h (Fig. 1). Neither PVP-I nor PHMB reduced bacterial cell density in 2-day *E. faecalis* ATCC 700802 biofilms after 0.5 h, but both did so after 3 and 6 h; after 24 h, the biofilms were eradicated.

For *S. aureus* ATCC BAA-43 (MRSA), PVP-I significantly reduced bacterial cell density compared with PHMB after 0.5 h (*P* = 0.0383), and both treatments completely eradicated the biofilm after 6 h. PVP-I demonstrated fast, potent anti-biofilm activity against *C. albicans* ATCC 10231, with complete biofilm eradication after 0.5 h; PHMB treatment resulted in complete eradication after 6 h.

### Test B: Anti-biofilm Activity of 10% PVP-I and 0.1% PHMB Against 3-Day, 5-Day, and 7-Day Biofilms of *P. aeruginosa* ATCC 15442

**Fig. 2:**
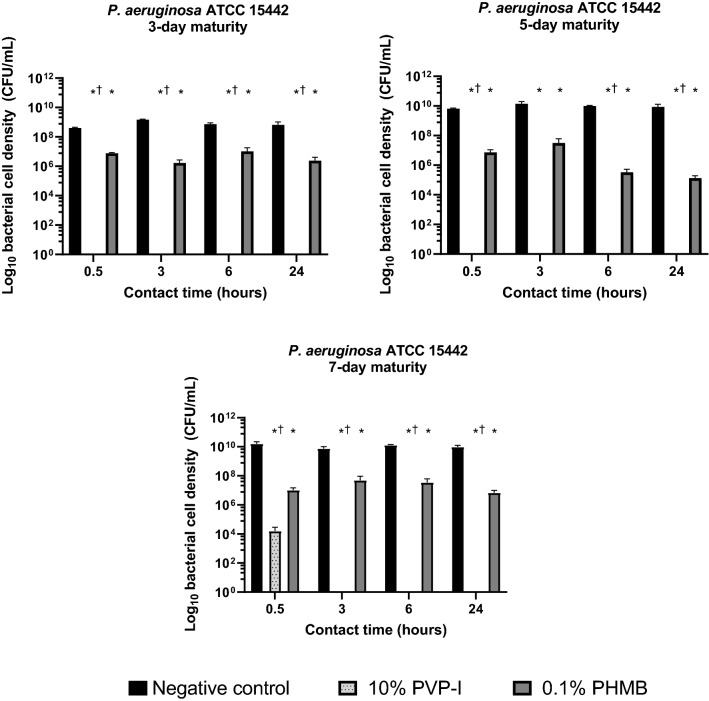
Bacterial cell density following treatment with PVP-I and PHMB in *P. aeruginosa* ATCC 15442 biofilms of 3-day maturity, 5-day maturity, and 7-day maturity. Log_10_ bacterial cell density (CFU/mL) of *P. aeruginosa* ATCC 15442 biofilms following 0.5, 3, 6, and 24-h treatment with 10% PVP-I and 0.1% PHMB (*n* = 3). Error bars represent standard error of the mean. *Significant reduction (*P* < 0.05) versus negative control. ^†^Significant reduction (*P* < 0.05) versus 0.1% PHMB. *CFU* colony-forming unit; *PHMB* polyhexamethylene biguanide; *PVP-I* povidone-iodine

PVP-I rapidly reduced bacterial cell density in 3-day, 5-day, and 7-day *P. aeruginosa* ATCC 15442 biofilms (Fig. 2). The 3-day and 5-day biofilms were completely eradicated after 0.5 h contact time with PVP-I, while the 7-day biofilm was completely eradicated after 3 h of PVP-I treatment.

PHMB significantly reduced bacterial cell density in *P. aeruginosa* ATCC 15442 biofilms of all maturities after just 0.5 h compared with the negative control (all *P* < 0.05), but did not result in complete biofilm eradication at any timepoint. PVP-I had significantly greater efficacy compared with PHMB at nearly all timepoints (*P* < 0.05), with the exception of the 3-h timepoint in the 5-day biofilm, in which an outlying value resulted in non-significance.

### Test C: Anti-biofilm Activity of 10% PVP-I and 0.1% PHMB Against 3-Day Biofilms of *S. aureus *BAA-43 (MRSA) and *S. aureus* ATCC 6538

**Fig. 3:**
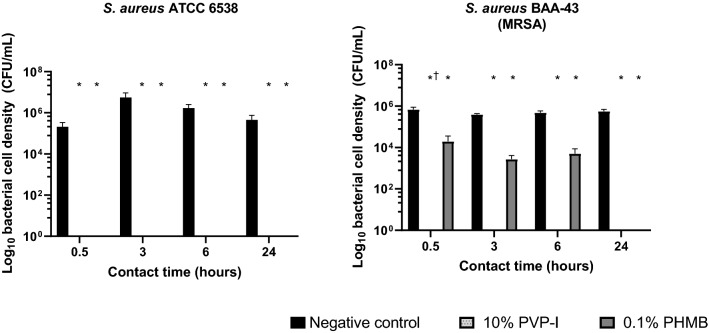
Bacterial cell density following treatment with PVP-I and PHMB in 3-day biofilms of *S. aureus* ATCC 6538 and *S. aureus* BAA-43 (MRSA). Log_10_ bacterial cell density (CFU/mL) of *S. aureus* biofilms following 0.5, 3, 6, and 24-h treatment with 10% PVP-I and 0.1% PHMB (*n* = 3). Error bars represent standard error of the mean. *Significant reduction (*P* < 0.05) versus negative control. ^†^Significant reduction (*P* = 0.027) versus 0.1% PHMB. *CFU* colony-forming unit; *MRSA* methicillin-resistant *S. aureus*; *PHMB* polyhexamethylene biguanide; *PVP-I* povidone-iodine

Compared with the negative control, both PVP-I and PHMB significantly reduced cell density in *S. aureus* ATCC 6538 and *S. aureus* ATCC BAA-43 (MRSA) 3-day biofilms at all timepoints (all *P* < 0.05). The data showed complete eradication of the *S. aureus* ATCC 6538 biofilm after 0.5 h with both antimicrobials (Fig. 3).

PVP-I completely eradicated the *S. aureus* ATCC BAA-43 (MRSA) biofilm after 0.5 h, and bacterial cell density was significantly reduced with PVP-I compared with PHMB at the 0.5-h timepoint (*P* = 0.0297). PHMB significantly reduced the *S. aureus* ATCC BAA-43 (MRSA) biofilm compared with the negative control at all timepoints (all *P* < 0.05) but did not result in complete eradication until the 24-h timepoint.

### Test D: 2D Time-Lapse Confocal Microscopy Images of *S. aureus* BAA-43 (MRSA) Biofilms Treated with 10% PVP-I and 0.1% PHMB

**Fig. 4:**
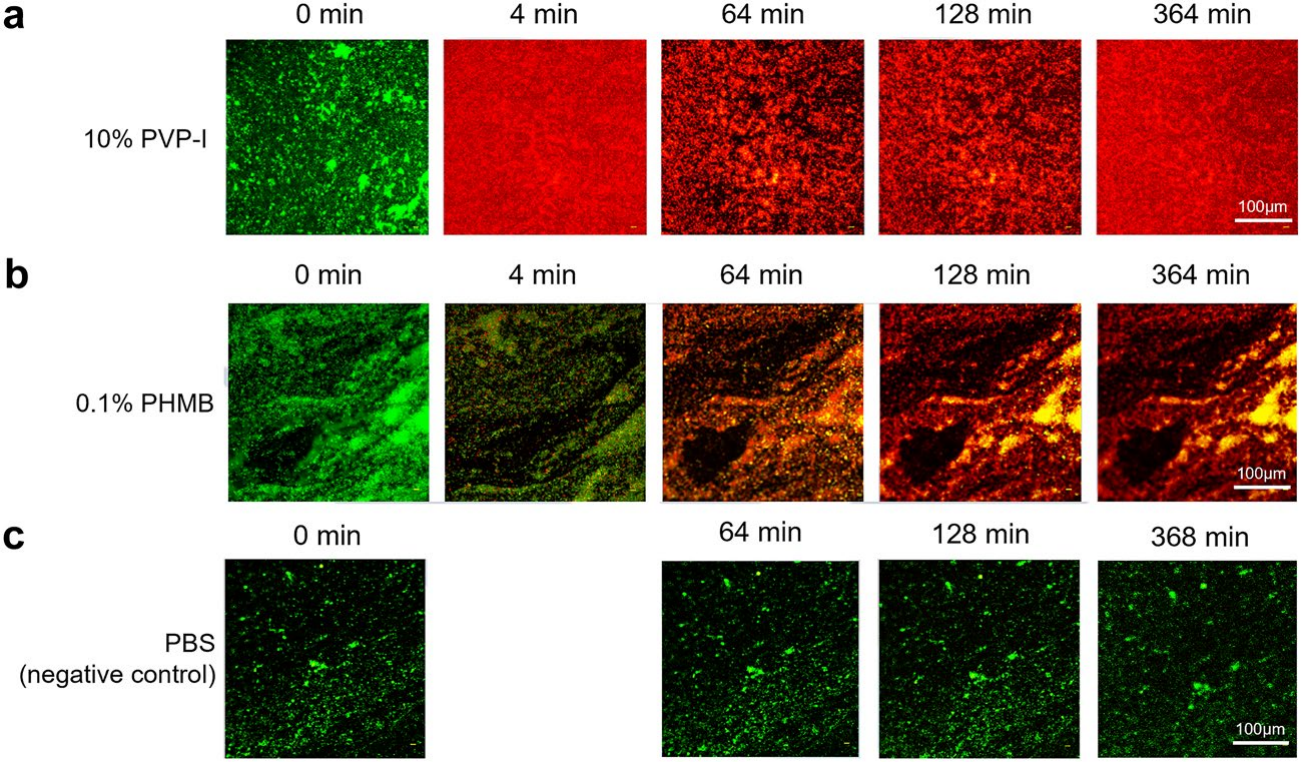
Live/dead composite confocal images of *S. aureus* BAA-43 (MRSA) 2-day biofilms treated with **a** 10% PVP-I, **b** 0.1% PHMB, and **c** PBS (negative control)*. Red is propidium iodide (dead stain) fluorescence; green is SYTO 9 (live stain) fluorescence. *The PVP-I- and PHMB-treated samples were imaged every 4 min; the PBS-treated sample was imaged every 16 min. *MRSA* methicillin-resistant *Staphylococcus aureus*; *PBS* phosphate-buffered saline; *PHMB* polyhexamethylene biguanide; *PVP-I* povidone-iodine

Confocal microscopy images with live/dead cell staining indicated rapid bactericidal activity with PVP-I after 4 min (Fig. 4a). The development of “gaps” in the biofilm after 64 min of PVP-I contact time suggests breakdown of EPS. When compared qualitatively with the PHMB-treated biofilms (Fig. 4b), PVP-I displayed a more rapid bactericidal activity than PHMB. No cell death was observed in the negative control (Fig. 4c).

## Discussion

Overall, this study confirms the in vitro anti-biofilm activity of PVP-I and PHMB against monomicrobial biofilms of representative Gram-negative bacteria, Gram-positive bacteria, antibiotic-resistant bacterial strains, and fungi. The results indicate that PVP-I has similar in vitro anti-biofilm activity to PHMB against various biofilms, and in some cases demonstrated more potent and rapid anti-biofilm activity versus PHMB, possibly in part due to PVP-I-mediated breakdown of EPS.

These findings suggest that PVP-I may be effective for suppressing biofilms in chronic wounds. This is concordant with previous pre-clinical publications: compared with silver-based foam dressings or control gauze, PVP-I 3% foam dressing was the most effective in wound healing by promoting neovascularization, re-epithelialization, and collagen deposition in an in vivo rat wound model [20]. Similarly, 10% PVP-I solution promoted rapid neovascularization more effectively than silver nitrate solution in an in vivo mouse wound model [21].

In vitro evidence suggests that PVP-I may facilitate wound healing by exerting an anti-inflammatory effect, scavenging superoxide anions, and inhibiting the production of reactive oxygen species by human polymorphonuclear neutrophils [22]. The anti-inflammatory action of PVP-I is further supported by a study of a rat acute skin wound model, in which PVP-I upregulated transforming growth factor beta, which suppresses the inflammatory response, and also increased neovascularization and re‐epithelialization [23]. An alternative mechanism was elucidated following a wound fluid analysis from patients with chronic non-healing venous leg ulcers: PVP-I reduced the activity of plasmin, neutrophil elastase, and metalloproteinases, which normally contribute to perturbation of tissue repair in chronic wounds [24].

Several clinical publications have reported the efficacy of PVP-I in favoring wound healing. PVP-I with hydrocolloid dressing improved venous leg ulcer healing rate compared with hydrocolloid dressing alone [25]. Another study demonstrated a significant improvement in chronic leg ulcers healing rate with PVP-I versus controls (silver sulfadiazine or chlorhexidine digluconate), reducing the time to healing by 2–9 weeks [26]. In patients undergoing split skin grafts, the use of PVP-I ointment medicated gauze did not delay wound healing compared with simple petrolatum gauze [27]. Evidence also suggested a possible earlier onset of epithelialization with PVP-I, and a trend toward lower bacterial counts versus petrolatum gauze controls [27]. Thus, there is a large body of evidence supporting the use of PVP-I in wound healing.

Biofilm management guidelines recommend a number of local antimicrobial agents following physical disruption of the biofilm and debridement, but do not recommend any one agent, due to a lack of clinical studies [11, 13]. There are many antimicrobial wound care products available, but prescribers’ ability to choose wound dressings is hindered by this lack of robust clinical- or cost-effectiveness data [28].

It may be prudent to consider the present findings, along with the body of evidence summarized above, for the development of any future biofilm or chronic wound management guidelines. Of note, we found PVP-I may have especially effective and rapid action against MRSA biofilms. In the microscopy images, the development of “gaps” in the PVP-I-treated MRSA biofilm suggests that PVP-I may contribute to the breakdown of biofilm EPS. Disrupting the EPS matrix using an EPS-targeting wound gel has been shown to improve wound healing outcomes [29]. Further study is required to examine the effects of PVP-I on EPS but our findings suggest that PVP-I may be effective in treating MRSA biofilm, partially due to EPS effects.

Our results support the algorithm for the management of chronic, non‐healing wounds, which recommends the use of PVP-I for mechanical washing, disinfection, and control of biofilm regrowth [8]. The authors suggest that, because of the rapid action of PVP‐I, a 1-min contact time may be sufficient to eradicate remaining microbes. The results presented here support this feature of PVP-I, demonstrating a more rapid action compared with PHMB against *P. aeruginosa* and MRSA. When using PHMB or other antimicrobials with slower onset of action [18], a longer contact time may be required; Andriessen and Strohal recommended a contact time of 10–15 min for PHMB due to its slow action [30].

Although the MBEC Assay and CDC Biofilm Reactor are standardized, accepted models for studying biofilms [19], some features of in vitro biofilm may not accurately reflect the characteristics of biofilms in clinical wounds [11]; it cannot be assumed that effective treatments for reducing biofilms in laboratory settings will have a similar impact in a wound [11]. Laboratory research may not reflect antimicrobial use in the clinic; for example, the contact time in this study was 0.5–24 h, while in the clinical setting an antimicrobial might remain in contact with the wound bed for 1–15 min (during wound cleansing and disinfection) [8, 11]. Finally, confocal microscopy depicts only one area of the chamber slide at one level; the possibility that the selected area may not represent the whole well or depth of the biofilms cannot be discounted. Thus, while in vitro studies can be insightful, there is a requirement for high-level research on the efficacy of topical antimicrobials to inform clinical decisions [11].

## Conclusion

In conclusion, the in vitro anti-biofilm activity of PVP-I is similar to that of PHMB, but with greater reduction of CFU count of mature *P. aeruginosa* ATCC 15442 biofilms and more rapid antimicrobial action against *S. aureus* ATCC BAA-43 (MRSA). This in vitro study contributes to the existing literature that supports the use of PVP-I for the treatment of chronic, non-healing wounds with biofilm, and suggests that PVP-I may be particularly effective in treating MRSA-associated biofilms. However, further high-quality, clinical research on the efficacy of antimicrobials is required.

## Data Availability

The datasets generated during and/or analyzed during the current study are not publicly available as data archiving is not mandated for in vitro studies. However, data are available from the corresponding author on reasonable request.
